# Disability-Related Disparities in Preventive Healthcare Access in South Korea: Insights From National Health Insurance Data

**DOI:** 10.3389/ijph.2025.1608644

**Published:** 2025-11-14

**Authors:** Sujin Kim, Boyoung Jeon

**Affiliations:** 1 Korea Institute for Health and Social Affairs, Sejong, Republic of Korea; 2 Myongji College, Seoul, Republic of Korea

**Keywords:** persons with disabilities, health inequities, health screening, accessibility, preventive healthcare

## Abstract

**Objectives:**

To examine disability-related disparities in participation in national health screenings in South Korea and to determine how these gaps vary by disability severity, type, and socio-economic factors.

**Methods:**

A trend analysis of screening participation from 2012 to 2020 and multivariable logistic regression for 2019–2020 were conducted using the National Health Insurance Service database, linking eligibility, health screening, and disability registration data. The cohort comprised 10,413,089 adults aged ≥40 years (20% population sample). Annual screening uptake was the outcome; predictors included disability status, severity, type, employment, sex, income, insurance, and region.

**Results:**

Screening uptake rose overall between 2012 and 2020 yet remained lower for people with disabilities, particularly those with severe physical, visual, communication, mental, developmental, epilepsy, and internal disabilities. Age-sex standardization and socio-economic adjustment attenuated but did not remove gaps. Employment narrowed disparities, whereas women with disabilities faced wider gaps than men.

**Conclusion:**

Despite nationwide gains, disability-related inequities in preventive screening persist, amplified by severe impairment, unemployment, and female gender. Policies should prioritize accessible facilities, targeted outreach, and socio-economic support to ensure equitable screening for people with disabilities.

## Introduction

Preventive services aim to reduce an individual’s risk of illness, disabilities, and death from preventable conditions [1]. Public health experts in high-income countries like the United Kingdom, the United States, and South Korea recommended preventive services such as cancer screening, behavioral counseling, and blood pressure and cholesterol checkups [2, 3]. Despite the documented benefits of utilizing preventive services in reducing disease incidence and premature mortality, these services are often underutilized by populations that need them the most. Ensuring equitable access to preventive care is especially crucial for persons with disabilities, who frequently experience poorer health outcomes due to systemic inequities within the healthcare system [4]. In addition, individuals with disabilities encounter health challenges from a young age and are susceptible to the same health issues as the general population as they age. Given this narrower margin of health, individuals with disabilities require access to a comprehensive care system, including preventive services, to mitigate the risks of illness, disability progression, and mortality from preventable conditions [5]. Despite this pressing need, however, individuals with disabilities often face significant barriers to accessing clinical preventive care, partly due to low prioritization and urgency within the healthcare system.

Previous studies have shown that the disabled population is less likely to receive health screenings, such as mammography and Pap tests, compared to the individuals without disabilities [5, 6]. In particular, subgroups such as adults with intellectual and physical disabilities face barriers in accessing cancer screening services [7–12] and regular health check-ups [13, 14]. Research on disability-related disparities has found high rates of delayed or forgone care due to higher un-insurance among persons with conditions such as mental disorders and low income [5, 15]. However, existing literature primarily relies on small self-reported samples [14], thus being limited by insufficient subdivision of disability types and severity levels and reporting errors and overlooking factors like the recommended service frequency. While several studies have utilized health claims data, their primary focus has been on cancer screening rates [8–12] or specific types of disabilities [7, 16, 17], or disparities among subgroups of individuals with disabilities without comparing them to the individuals without disabilities [18, 19]. Given the increasing burden of cardiovascular disease among persons with disabilities and the recommendations for regular health checkups, including blood pressure and cholesterol screenings [2, 3], access to these preventive care services should be examined.

In South Korea, the National Health Insurance Service (NHIS) provides all citizens aged 20 years or older with the General Healthcare Screening Program (GHSP) to reduce the risks of cardio-cerebrovascular diseases. Since nearly all South Koreans are insured through NHIS, GHSP is accessible either for free or at a minimal cost, minimizing financial barriers to participation [20]. Nevertheless, healthcare disparities persist for individuals with disabilities [12, 21, 22]. To address these disparities, the South Korean government has designated and financially supported health screening centers specifically for individuals with disabilities since 2018 [23]. Furthermore, since 2019, additional subsidies have been allocated to institutions providing health screenings to those with severe disabilities [3]. Despite these initiatives, recent changes in disability-related disparities in health screenings have yet to be fully examined. To further close these gaps in screening participation and foster inclusivity and equitable access, it is imperative to examine current trends and explore the roles of socio-economic status, such as employment, and sex differences in shaping disability-related disparities. The NHIS database in South Korea contains comprehensive health screening data and socio-economic information for the entire population, as well as detailed records of disability type and severity based on national disability registration criteria [24]. These unique data features offer a valuable opportunity to systematically assess the impact of different disability types and severity levels on healthcare disparities.

In this study, we linked GHSP data, eligibility data and disability registration datasets from the National Health Insurance Service to address two objectives. The first objective was to determine the variation in GHSP participation rates over time based on the presence or absence of a disability (hereafter referred to as disability status). We further elucidated these temporal trends among individuals with disabilities considering the type and severity of the disabilities. The second objective was to examine the association between disability status and health screening participation during the years 2019–2020, focusing on the role of socio-economic factors, like employment status, and differences by sex, by stratifying the data accordingly.

## Methods

### Data Sources

#### National Health Insurance Service (NHIS) Database

NHIS provides mandatory healthcare coverage for virtually all citizens of South Korea, which is around 50 million people. This service includes NHI enrollees (97%) and medical aid beneficiaries (3%) and covers most forms of healthcare services, such as emergency room visits, inpatient and outpatient care, and medication prescriptions. NHI enrollees are individuals who pay monthly insurance contributions, whereas medical aid beneficiaries are individuals from low-income households supported by the government’s Medical Aid Program and are exempt from contributions. Accordingly, the NHIS database consists of sociodemographic and clinical information on medical claims.

#### General Healthcare Screening Program (GHSP)

The GHSP is a national preventive screening program provided to eligible individuals to detect major chronic and cardiovascular diseases. While nearly all citizens are covered by national health insurance, eligibility for the GHSP is determined by age and insurance classification [20]. Specific eligibility criteria vary by insurance classification. NHI enrollees, such as employed or self-employed individuals, are eligible for biennial screenings, with an exception for those in manual labor who are eligible annually. For other groups, dependents of NHI enrollees aged 40 and older, along with medical aid beneficiaries aged 40–64, are also entitled to biennial screenings. Since 2019, eligibility for these latter two groups was expanded to include individuals aged 20 to 39. The program primarily screens for cardio-cerebrovascular diseases, including hypertension, diabetes mellitus, dyslipidemia, and obesity. Additional tests are provided based on the participant’s sex and age (e.g., osteoporosis screening for women at 66 years of age) [20].

### Disability Registration System and Definition of Disability Types

The South Korean government established a national disability registration system to provide appropriate welfare benefits, which are determined based on the type and severity of the disability. This system recognizes 15 types of disabilities and divides them into six severity levels: Grade 1 being the “most severe” and Grade 6 being the “least severe.” In addition, the broad categorization of severity levels specifies Grades 1 to 3 as “severe” and Grades 4 to 6 as “moderate.” The severity levels are determined by physician specialists based on the degree of functional loss and clinical impairment, following criteria defined by the Ministry of Health and Welfare guidelines [25].

In this study, we based our classification on the 15 official disability types recognized by South Korea’s Welfare of Disabled Persons Act: physical disability, brain injury, visual impairment, hearing impairment, speech impairment, facial disability, kidney disability, heart disease, liver disease, respiratory disorders, colostomy/urostomy, epilepsy, intellectual disability, autism, and mental disability [25]. For our analysis, these 15 types were reclassified into nine final analytical groups (one non-disability group and eight broader disability categories). Several of these new categories were defined by combining related impairments: Physical disability was defined to encompass limb and brain lesions; Communication disability included hearing and speech impairments; Developmental/Intellectual disability covered intellectual disability and autism spectrum disorders; and Internal disability grouped kidney, heart, liver, respiratory, and digestive disorders. The final categories used in the study, stratified by severity where applicable, were: No disability, Physical disability (moderate and severe), Visual disability (moderate and severe), Communication disability (moderate and severe), Mental disability, Developmental/Intellectual disability, Facial disability (moderate and severe), Epilepsy disability (moderate and severe), and Internal disability (moderate and severe) (Supplementary Table S1).

Each category was further stratified by severity, enabling a detailed examination of the relationship between disability type, severity, and access to preventive healthcare. For individuals with multiple impairments, the national registration system has a process for combining disabilities to determine a final, single severity grade. According to the official guidelines, this combination is permitted only when it results in a more severe overall disability level, with specific criteria varying by disability type [26]. Because the final administrative data registers each individual under a single classification, the independent effects of co-occurring disabilities could not be analyzed in this study. Furthermore, the classification of severity differs significantly by disability type. According to Korea’s Welfare of Disabled Persons Act, mental and developmental (i.e., intellectual and autistic) disabilities are only registered if the impairment is classified as “severe”; a “non-severe” (mild) grade does not legally exist for these categories in the registration system [25].

### Study Population

In total, 10,413,089 participants from the 2012 NHIS database, representing approximately 20% of the entire population, were randomly selected based on sex, age (birth year), and region of residence (municipal districts with si-gun-gu level) and were then followed up until December 31, 2021, unless their eligibility was revoked due to death or emigration (NHIS-2022-1-629). Access to the full population-level dataset is restricted for researchers under the data-sharing policy of the National Health Insurance Service (NHIS); therefore, this study utilized a representative sample cohort provided by the agency. Using this sample database, we constructed a repeated cross-sectional dataset of adults aged 40 years or older who were eligible for GHSP in each year from 2012 to 2021. We restricted the study population to individuals aged 40 years or older to ensure consistent eligibility criteria across the entire trend analysis period; eligibility for many population groups, such as dependents of the insured, only began at age 40 prior to a major policy expansion in 2019 that included younger adults [27]. For the regression analyses of the 2019–2020 data, a total of 88,488 participants (1.4%) were excluded due to missing income information. As anonymized data were provided by NHIS under strict confidentiality guidelines, the study was exempt from ethics review by the Institutional Review Board of the Korea Institute for Health and Social Affairs (IRB number: 2022-004).

### Outcome Measures

First, we calculated crude participation rates, defined as the proportion of eligible individuals who received GHSP among the target population. To account for differences in population structure, age- and sex-standardized participation rates were estimated using the direct standardization method with the 2019 South Korean resident population as the standard, stratified by age (40–49, 50–64, ≥65 years). For regression analyses, we modeled non-participation in health screening as a binary outcome (1 = did not participate; 0 = participated) using logistic regression. This approach was chosen to directly model the negative outcome of non-participation, which highlights barriers to access and is often considered more relevant for policy interventions aimed at reducing health disparities.

### Statistical Analyses

To address Objective 1, using the nine analytical groups described in the “Disability registration system and definition of disability types” section (one non-disability group and eight broader disability categories), we examined the yearly crude and age- and sex-standardized participation rates for each group from 2012 to 2020. To address Objective 2, we then conducted multivariate logistic regression analyses using the combined 2019–2020 dataset to examine factors influencing GHSP non-participation.

Because the GHSP is offered on a biennial basis for most participants, data from 2019 to 2020 were pooled to capture a full screening cycle and provide a more representative sample of the eligible population. For these regression analyses, each person-year observation was treated as an independent unit to assess the overall association between disability and screening non-participation during this period. We developed two nested models. Model 1 assessed the association between disability status and screening non-participation, adjusting for core demographic variables including age (as a continuous variable with its square term), sex, and year. Model 2 added key sociodemographic variables to Model 1, including healthcare coverage type (NHI employee or regional), income level (quintiles), and region of residence.

The disability groups included no disability, physical disability (moderate and severe), visual disability (moderate and severe), communication disability (moderate and severe), mental disability, developmental/intellectual disability, facial disability (moderate and severe), epilepsy disability (moderate and severe), and internal disability (moderate and severe). Age was used as a continuous variable, including its square term to account for potential nonlinear effects. Healthcare coverage was categorized into National Health Insurance (NHI) enrollees (subdivided into employee enrollees, who are salaried workers, and regional enrollees, who are all other enrollees such as the self-employed, freelancers, and retirees) and medical aid beneficiaries. Participants’ income levels were classified into five quintiles, with medical aid beneficiaries as the first contribution quintile, followed by the second, third, fourth, and fifth quintiles in ascending order of income. The regions of residence were categorized as metropolitan areas, cities, and rural areas.

We first compared screening rates across different types and levels of disability, adjusting only for age and sex (Models 1, 3, and 5 of Table 3). We then added additional covariates, including health coverage type, income quintiles, and region of residence (Models 2, 4, and 6 of Table 3 and all models of Table 4). All analytical models included a year variable (2019 vs. 2020, with 2020 as the reference) to account for potential period effects associated with the COVID-19 pandemic. The resulting Odds Ratios (ORs) were interpreted as follows: an OR significantly greater than 1 indicated a higher likelihood of non-participation (a negative disparity), while an OR significantly less than 1 indicated a lower likelihood of non-participation (a positive disparity or advantage) compared to the non-disability group. Data analyses were performed using SAS Enterprise Guide 7.1 (SAS Institute, Cary, NC, USA).

## Results

### Study Participants

The number of individuals invited to undergo GHSP increased from 2,519,058 in 2012 to 3,028,007 in 2020. Among them, the proportion of participants with a registered disability decreased from 7.88% in 2012 to 7.10% in 2020 (Table 1).

**TABLE 1:** General Healthcare Screening Program participation rates across different disability groups (South Korea, 2012–2020).

Disability status		2012	2013	2014	2015	2016	2017	2018	2019	2020
No disability	Eligible individuals	2,320,467	2,376,618	2,489,424	2,625,537	2,673,651	2,710,590	2,819,745	2,869,132	2,813,119
	Screening	1,633,322	1,650,547	1,795,517	1,846,781	1,932,549	1,981,063	2,115,173	2,188,916	1,961,858
	Crude rate	70.39	69.45	72.13	70.34	72.28	73.09	75.01	76.29	69.74
Any disability	Eligible individuals	198,591	200,527	203,676	209,338	212,943	213,676	232,206	221,033	214,888
	Screening	126,147	126,281	132,832	132,071	136,907	137,110	147,346	144,964	126,044
	Crude rate	63.52	62.97	65.22	63.09	64.29	64.17	63.45	65.58	58.66
Physical disability, moderate	Eligible individuals	98,392	99,370	101,611	104,227	106,118	104,440	110,402	103,942	100,110
	Screening	70,194	69,841	73,186	72,780	75,548	74,259	78,525	76,040	66,383
	Crude rate	71.34	70.28	72.03	69.83	71.19	71.10	71.13	73.16	66.31
Physical disability, severe	Eligible individuals	34,930	34,225	33,971	33,733	33,708	32,708	35,200	31,848	30,402
	Screening	18,381	17,836	18,661	17,742	18,291	17,541	18,523	17,322	14,491
	Crude rate	52.62	52.11	54.93	52.60	54.26	53.63	52.62	54.39	47.66
Visual disability, moderate	Eligible individuals	16,215	16,644	17,035	17,965	18,043	18,157	19,448	18,654	17,975
	Screening	11,108	11,234	11,909	12,183	12,409	12,458	13,364	13,260	11,626
	Crude rate	68.50	67.50	69.91	67.82	68.77	68.61	68.72	71.08	64.68
Visual disability, severe	Eligible individuals	3,886	3,835	3,819	3,763	3,804	3,725	4,260	3,696	3,539
	Screening	2,095	2,033	2,131	2,085	2,114	2,085	2,292	2,163	1,783
	Crude rate	53.91	53.01	55.80	55.41	55.57	55.97	53.80	58.52	50.38
Communication disability, moderate	Eligible individuals	14,273	14,244	14,540	15,073	16,616	19,167	24,429	25,753	26,505
	Screening	9,281	9,131	9,566	9,707	10,850	12,470	15,308	16,862	15,279
	Crude rate	65.02	64.10	65.79	64.40	65.30	65.06	62.66	65.48	57.65
Communication disability, severe	Eligible individuals	8,156	8,125	8,149	8,187	8,309	8,363	9,218	8,449	7,881
	Screening	4,749	4,728	4,889	4,812	4,966	5,037	5,291	5,229	4,266
	Crude rate	58.23	58.19	60.00	58.78	59.77	60.23	57.40	61.89	54.13
Mental disability	Eligible individuals	5,247	5,690	5,998	6,408	6,936	7,184	7,851	7,772	7,892
	Screening	2,387	2,877	3,265	3,239	3,510	3,689	3,967	4,003	3,540
	Crude rate	45.49	50.56	54.43	50.55	50.61	51.35	50.53	51.51	44.86
Developmental/Intellectual disability	Eligible individuals	7,572	7,995	8,040	8,432	8,494	8,623	9,196	8,652	8,328
	Screening	3,215	3,607	3,920	3,829	3,916	3,982	4,156	3,957	3,233
	Crude rate	42.46	45.12	48.76	45.41	46.10	46.18	45.19	45.74	38.82
Facial disability, moderate	Eligible individuals	146	176	151	190	93	99	100	112	96
	Screening	108	136	110	151	64	76	72	90	73
	Crude rate	73.97	77.27	72.85	79.47	68.82	76.77	72.00	80.36	76.04
Facial disability, severe	Eligible individuals	94	94	105	109	113	117	125	130	120
	Screening	56	67	67	71	69	83	86	98	72
	Crude rate	59.57	71.28	63.81	65.14	61.06	70.94	68.80	75.38	60.00
Epilepsy disability, moderate	Eligible individuals	440	486	456	522	488	487	462	482	435
	Screening	250	310	302	321	291	295	275	285	238
	Crude rate	56.82	63.79	66.23	61.49	59.63	60.57	59.52	59.13	54.71
Epilepsy disability, severe	Eligible individuals	262	240	223	196	155	135	154	140	140
	Screening	159	133	142	116	82	72	78	81	63
	Crude rate	60.69	55.42	63.68	59.18	52.90	53.33	50.65	57.86	45.00
Internal disability, moderate	Eligible individuals	3,082	3,287	3,548	3,934	3,575	3,807	4,189	4,348	4,324
	Screening	1,704	1,783	2,019	2,180	1,967	2,171	2,378	2,565	2,332
	Crude rate	55.29	54.24	56.91	55.41	55.02	57.03	56.77	58.99	53.93
Internal disability, severe	Obs.	5896	6116	6030	6599	6491	6664	7172	7055	7141
	Screening	2460	2565	2665	2855	2830	2892	3031	3009	2665
	Crude rate	41.72	41.94	44.20	43.26	43.60	43.40	42.26	42.65	37.32

The Crude rate is presented as a percentage (per 100 eligible individuals).

### Trends in Health Screening Rates According to Disability Status

Table 1 shows the crude rates of health screening over 9 years, subdivided by disability status and type. Although the overall rate showed an increasing trend over time, except in 2020, the rates of health screening were relatively low among participants with disabilities, particularly those with severe internal, mental, developmental, or severe epilepsy disabilities. In contrast, the rates for those with moderate physical and moderate facial disabilities were comparable (Table 1). The overall gap between participants with and without disabilities decreased after sex-age standardization (Supplementary Table S2). These nine-year trends are visually represented in Supplementary Figure S1.

### Factors Associated With Health Screening Rates

Table 2 presents the characteristics of the study samples from 2019 to 2020. The average age of participants without disabilities was 56.5 years, which was lower than that of participants with physical, visual, hearing/speech, or internal disabilities. Among participants with disabilities, 30.2% were in the highest income quintile, whereas 18.9% were in the lowest income quintile, with individuals having intellectual and developmental, mental, or epilepsy disabilities concentrated in this lowest quintile. The proportion of participants without disabilities receiving medical aid was 1.2%, which was lower than that of participants with disabilities. Notably, medical aid beneficiaries were concentrated among participants with epilepsy (moderate 46.7%; severe 49.5%), intellectual or developmental disabilities (60.7%), and mental disabilities (66.9%) (Table 2).

**TABLE 2:** Descriptive statistics of study samples (South Korea, 2012–2020).

	No disability	Physical disability		Visual disability		Communication disability		Developmental/intellectual disability		Facial disability		Epilepsy		Internal disability		*p-value*
		Moderate	Severe	Moderate	Severe	Moderate	Severe	Moderate	Severe	Moderate	Severe	Moderate	Severe	Moderate	Severe	
N	5,599,615	201,128	61,337	36,162	7,156	51,462	16,109	15,606	16,902	206	246	911	279	8,555	14,010	
Age, year	56.47	64.94	64.26	64.77	66.43	74.18	67.75	51.51	54.26	55.65	56.71	54.17	54.63	61.10	63.93	
(SD)	(11.4)	(11.9)	(12.1)	(12.6)	(13.4)	(11.5)	(13.5)	(8.4)	(8.0)	(11.6)	(11.1)	(8.1)	(9.3)	(11.0)	(11.5)	
Men	2,771,122	119,003	39,236	22,828	3,719	28,622	9,119	9,036	8,791	116	142	476	151	5,593	8,852	***
(%)	(49.5)	(59.2)	(64.0)	(63.1)	(52.0)	(55.6)	(56.6)	(57.9)	(52.0)	(56.3)	(57.7)	(52.3)	(54.1)	(65.4)	(63.2)	
Women	2,828,493	82,125	22,101	13,334	3,437	22,840	6,990	6,570	8,111	90	104	435	128	2,962	5,158	
(%)	(50.5)	(40.8)	(36.0)	(36.9)	(48.0)	(44.4)	(43.4)	(42.1)	(48.0)	(43.7)	(42.3)	(47.7)	(45.9)	(34.6)	(36.8)	
Medical aid	65,365	9,109	10,111	1,535	1,297	1,086	1,637	9,480	11,300	20	31	425	138	658	2,469	***
(%)	(1.2)	(4.5)	(16.5)	(4.2)	(18.1)	(2.1)	(10.2)	(60.7)	(66.9)	(9.7)	(12.6)	(46.7)	(49.5)	(7.7)	(17.6)	
NHI, region	2,750,063	128,920	39,083	23,101	4,637	41,564	10,940	4,207	4,722	82	106	365	107	4,940	9,423	
(%)	(49.1)	(64.1)	(63.7)	(63.9)	(64.8)	(80.8)	(67.9)	(27.0)	(27.9)	(39.8)	(43.1)	(40.1)	(38.4)	(57.7)	(67.3)	
NHI, employee	2,784,187	63,099	12,143	11,526	1,222	8,812	3,532	1,919	880	104	109	121	34	2,957	2,118	
(%)	(49.7)	(31.4)	(19.8)	(31.9)	(17.1)	(17.1)	(21.9)	(12.3)	(5.2)	(50.5)	(44.3)	(13.3)	(12.2)	(34.6)	(15.1)	
Lowest income	1,061,017	45,160	20,407	8,385	2,772	10,275	4,530	11,826	13,160	49	85	567	176	2,033	4,694	***
(%)	(18.9)	(22.5)	(33.3)	(23.2)	(38.7)	(20.0)	(28.1)	(75.8)	(77.9)	(23.8)	(34.6)	(62.2)	(63.1)	(23.8)	(33.5)	
2nd income	773,099	25,428	6,530	5,022	732	5,882	1,910	897	734	24	40	94	30	949	1,411	
(%)	(13.8)	(12.6)	(10.6)	(13.9)	(10.2)	(11.4)	(11.9)	(5.7)	(4.3)	(11.7)	(16.3)	(10.3)	(10.8)	(11.1)	(10.1)	
3rd income	926,431	32,039	8,619	5,814	862	7,373	2,522	978	916	45	39	90	31	1,230	1,967	
(%)	(16.5)	(15.9)	(14.1)	(16.1)	(12.0)	(14.3)	(15.7)	(6.3)	(5.4)	(21.8)	(15.9)	(9.9)	(11.1)	(14.4)	(14.0)	
4th income	1,147,279	39,901	10,836	6,977	1,087	9,487	2,974	963	932	49	43	88	20	1,580	2,413	
(%)	(20.5)	(19.8)	(17.7)	(19.3)	(15.2)	(18.4)	(18.5)	(6.2)	(5.5)	(23.8)	(17.5)	(9.7)	(7.2)	(18.5)	(17.2)	
Highest income	1,691,789	58,600	14,945	9,964	1,703	18,445	4,173	942	1,160	39	39	72	22	2,763	3,525	
(%)	(30.2)	(29.1)	(24.4)	(27.6)	(23.8)	(35.8)	(25.9)	(6.0)	(6.9)	(18.9)	(15.9)	(7.9)	(7.9)	(32.3)	(25.2)	
Metropolitan	996,731	28,635	8,945	5,648	1,353	7,292	2,202	1,786	2,692	31	31	160	53	1,557	2,613	***
(%)	(17.8)	(14.2)	(14.6)	(15.6)	(18.9)	(14.2)	(13.7)	(11.4)	(15.9)	(15.0)	(12.6)	(17.6)	(19.0)	(18.2)	(18.7)	
Cities	1,453,950	49,506	15,275	9,360	1,761	13,245	3,898	3,343	4,497	50	78	261	78	2,189	3,783	
(%)	(26.0)	(24.6)	(24.9)	(25.9)	(24.6)	(25.7)	(24.2)	(21.4)	(26.6)	(24.3)	(31.7)	(28.6)	(28.0)	(25.6)	(27.0)	
Rural	3,148,934	122,987	37,117	21,154	4,042	30,925	10,009	10,477	9,713	125	137	490	148	4,809	7,614	
(%)	(56.2)	(61.1)	(60.5)	(58.5)	(56.5)	(60.1)	(62.1)	(67.1)	(57.5)	(60.7)	(55.7)	(53.8)	(53.0)	(56.2)	(54.3)	
2019	2,827,841	102,396	31,355	18,416	3,655	25,361	8,332	7,739	8,613	110	130	480	140	4,297	6,964	***
(%)	(50.5)	(50.9)	(51.1)	(50.9)	(51.1)	(49.3)	(51.7)	(49.6)	(51.0)	(53.4)	(52.8)	(52.7)	(50.2)	(50.2)	(49.7)	
2020	2,771,774	98,732	29,982	17,746	3,501	26,101	7,777	7,867	8,289	96	116	431	139	4,258	7,046	
(%)	(49.5)	(49.1)	(48.9)	(49.1)	(48.9)	(50.7)	(48.3)	(50.4)	(49.0)	(46.6)	(47.2)	(47.3)	(49.8)	(49.8)	(50.3)	

*** <0.001, ** <0.01, *<0.05. NHI means National Health Insurance. N represents the total sample size for each group in the 2019–2020 combined dataset.

Models 1 and 2 in Table 3 show the association between disability status and health screening rates. In Model 1, the following subgroups of individuals with disabilities exhibited higher odds of not receiving health screening than individuals without disabilities, in descending order of Odds Ratios (ORs): developmental/intellectual (OR: 4.19, 95% CI: 4.14–4.25), severe internal (OR: 3.83, 95% CI: 3.77–3.89) mental (OR: 3.19, 95% CI: 3.15–3.24), severe epilepsy (OR: 2.75, 95% CI: 2.47–3.06), severe physical (OR: 2.33, 95% CI: 2.31–2.34), moderate epilepsy (OR: 2.31, 95% CI: 2.18–2.46), severe visual (OR: 1.78, 95% CI: 1.74–1.82), and severe communication disabilities (OR: 1.45, 95% CI: 1.43–1.47). Conversely, moderate physical, hearing, speech, and facial disability subgroups did not show higher odds compared to those without disabilities. In Model 2, which additionally adjusted for health coverage type, income quintiles, and region of residence, results were similar to those in Model 1. However, in this model, the moderate visual and epilepsy disability subgroups were no longer significantly from those without disabilities. Moreover, the overall ORs were lower in Model 2 than in Model 1.

**TABLE 3:** Factors associated with non-participation in health screening (South Korea, 2012–2020).

	All (N = 6,029,684)		Employees (N = 3,136,921)		Others (NHI regional enrollees and medical aid beneficiaries) (N = 2,892,763)	
	Model 1	Model 2	Model 3	Model 4	Model 5	Model 6
	Odds ratio (95% CI)	Odds ratio (95% CI)	Odds ratio (95% CI)	Odds ratio (95% CI)	Odds ratio (95% CI)	Odds ratio (95% CI)
Types of disability (ref. = No disability)						
Physical disability, moderate	0.993 (0.989, 0.998)	0.880 (0.876, 0.884)	0.842 (0.834, 0.851)	0.824 (0.816, 0.833)	0.957 (0.952, 0.962)	0.906 (0.901, 0.911)
Physical disability, severe	2.326 (2.309, 2.344)	1.632 (1.619, 1.644)	1.210 (1.186, 1.235)	1.122 (1.099, 1.145)	2.096 (2.078, 2.113)	1.776 (1.761, 1.791)
Visual disability, moderate	1.066 (1.055, 1.077)	0.937 (0.927, 0.947)	0.934 (0.913, 0.955)	0.899 (0.879, 0.920)	1.010 (0.998, 1.023)	0.959 (0.948, 0.971)
Visual disability, severe	1.783 (1.744, 1.823)	1.222 (1.195, 1.250)	1.015 (0.949, 1.087)	0.810 (0.756, 0.868)	1.591 (1.553, 1.629)	1.302 (1.271, 1.334)
Communication, moderate	0.969 (0.961, 0.978)	0.899 (0.891, 0.907)	0.791 (0.770, 0.813)	0.766 (0.745, 0.787)	0.934 (0.926, 0.943)	0.908 (0.899, 0.916)
Communication, severe	1.446 (1.425, 1.468)	1.163 (1.145, 1.181)	0.868 (0.832, 0.906)	0.818 (0.784, 0.853)	1.399 (1.376, 1.422)	1.244 (1.224, 1.265)
Mental disability	3.192 (3.147, 3.238)	1.106 (1.089, 1.123)	1.053 (0.998, 1.110)	0.751 (0.712, 0.793)	2.022 (1.991, 2.054)	1.112 (1.094, 1.131)
Developmental/Intellectual	4.192 (4.135, 4.251)	1.376 (1.356, 1.397)	0.903 (0.830, 0.982)	0.652 (0.599, 0.710)	2.615 (2.578, 2.653)	1.425 (1.403, 1.447)
Facial disability, moderate	0.753 (0.648, 0.876)	0.667 (0.570, 0.781)	0.415 (0.300, 0.575)	0.402 (0.290, 0.558)	0.952 (0.791, 1.147)	0.825 (0.683, 0.996)
Facial disability, severe	1.252 (1.107, 1.416)	1.015 (0.892, 1.155)	1.004 (0.798, 1.262)	0.963 (0.765, 1.212)	1.269 (1.086, 1.483)	1.048 (0.895, 1.226)
Epilepsy disability, moderate	2.314 (2.180, 2.455)	0.945 (0.888, 1.006)	0.574 (0.439, 0.752)	0.476 (0.363, 0.625)	1.595 (1.496, 1.700)	0.997 (0.934, 1.065)
Epilepsy disability, severe	2.750 (2.472, 3.059)	1.104 (0.987, 1.235)	0.319 (0.167, 0.608)	0.274 (0.144, 0.523)	1.957 (1.744, 2.195)	1.205 (1.071, 1.356)
Internal disability, moderate	2.074 (2.033, 2.115)	1.781 (1.745, 1.818)	1.940 (1.871, 2.012)	1.918 (1.848, 1.990)	1.865 (1.820, 1.911)	1.717 (1.676, 1.760)
Internal disability, severe	3.827 (3.767, 3.887)	2.586 (2.544, 2.628)	2.401 (2.304, 2.503)	2.231 (2.139, 2.326)	3.181 (3.127, 3.237)	2.728 (2.680, 2.776)
Sex (ref. = Men)						
Women	1.041 (1.039, 1.043)	0.823 (0.821, 0.824)	0.881 (0.879, 0.884)	0.693 (0.691, 0.695)	0.811 (0.809, 0.813)	0.821 (0.819, 0.823)
Age	0.847 (0.847, 0.848)	0.832 (0.832, 0.833)	0.950 (0.949, 0.952)	0.981 (0.979, 0.983)	0.779 (0.779, 0.780)	0.777 (0.777, 0.778)
Age square	1.002 (1.001, 1.002)	1.002 (1.002, 1.002)	1.000 (1.000, 1.001)	1.000 (1.000, 1.000)	1.002 (1.002, 1.002)	1.002 (1.002, 1.002)
Healthcare coverage (ref. = NHI, employees)						
NHI, region		3.339 (3.333, 3.346)		-		1.000
Medical aid		6.078 (6.040, 6.116)		-		2.066 (2.052, 2.079)
Income level (ref. = Highest income)						
Lowest income		1.484 (1.481, 1.488)		2.143 (2.133, 2.152)		1.190 (1.186, 1.194)
2nd income		1.102 (1.099, 1.105)		1.527 (1.519, 1.534)		0.933 (0.930, 0.937)
3rd income		1.038 (1.035, 1.040)		1.345 (1.339, 1.352)		0.936 (0.933, 0.939)
4th income		0.943 (0.940, 0.945)		1.074 (1.069, 1.078)		0.916 (0.913, 0.919)
Regions of residence (ref. = Metropolitan)						
Cities		0.776 (0.774, 0.778)		0.621 (0.619, 0.624)		0.879 (0.876, 0.882)
Rural		0.832 (0.830, 0.834)		0.705 (0.702, 0.707)		0.915 (0.913, 0.918)
Year (ref. = 2020)	0.714 (0.713, 0.716)	0.678 (0.677, 0.680)	0.622 (0.621, 0.624)	0.613 (0.611, 0.615)	0.714 (0.712, 0.715)	0.712 (0.711, 0.714)

The analytic models included year-fixed effects. NHI, means National Health Insurance. ref means reference.

Models 1, 3, 5: Adjusted for age (as a continuous variable with its square term), sex, and year (2019 vs. 2020).

Models 2, 4, 6: Adjusted for all variables in Models 1, 3, 5, plus healthcare coverage type, income level (quintiles), and region of residence.

Models 3 and 4 in Table 3 illustrate the association between disability status and health screening rates among employed participants. In Model 3, the following subgroups of participants with disabilities demonstrated higher odds of not receiving health screening than did individuals without disabilities: severe (OR: 2.59, 95% CI: 2.54–2.63) and moderate internal (OR: 1.78, 95% CI: 1.75–1.82), severe physical (OR: 1.63, 95% CI: 1.62–1.64), and developmental/intellectual (OR: 1.38, 95% CI: 1.36–1.40). The other subgroups of participants with disabilities did not show higher odds compared to individuals without disabilities. In Model 4, which additionally adjusted for health coverage type, income quintiles, and region of residence, the internal and severe physical disability subgroups had higher odds than individuals without disabilities.

The associations between disability status and health screening rates among others (NHI regional enrollees and Medical Aid beneficiaries) depicted in Models 5 and 6 were similar to those in Models 1 and 2, respectively. When adjusted only for sex and age, the following subgroups of individuals with disabilities showed higher odds than those without disabilities: severe internal (OR: 3.18, 95% CI: 3.13–3.24), mental (OR: 2.02, 95% CI: 1.99–2.05), severe physical (OR: 2.10, 95% CI: 2.08–2.11), developmental/intellectual (OR: 2.62, 95% CI: 2.58–2.65, severe (OR: 1.96, 95% CI: 1.74–2.19) and moderate epilepsy (OR: 1.59, 95% CI: 1.50–1.70), severe visual (OR: 1.59, 95% CI: 1.55–1.63), severe communication (OR: 1.40, 95% CI: 1.38–1.42), and severe facial disabilities (OR: 1.27, 95% CI: 1.09–1.48) (Table 3).

Table 4 presents the sex-stratified analysis. Overall, the disability-related disparities were more pronounced among women than men, with higher odds of non-participation (Models 1-6 in Table 4). In particular, women with severe epilepsy (OR: 1.26, 95% CI: 1.07–1.49 in Model 1) or developmental/intellectual disorders (OR:1.64, 95% CI: 1.61–1.68 in Model 1) showed higher odds compared to their male counterparts (OR: 1.00, 95% CI 0.86–1.17 and OR: 1.15, 95% CI 1.13–1.17 in Model 1, respectively). In contrast, men with these respective disabilities had lower or comparable odds of non-participation than their female counterparts (Models 1 and 2 in Table 4). Although employed women with severe physical disabilities did not have a significantly higher odds than their male counterparts, a notable difference was observed (p-value = 0.067) (Models 3 and 4 in Table 4).

**TABLE 4:** Factors associated with non-participation in health screening by sex (South Korea, 2012–2020).

	All		Employees		Others (NHI regional enrollees and medical aid beneficiaries)	
	Men (N = 3,026,806)	Women (N = 3,002,878)	Men (N = 1,752,519)	Women (N = 1,140,244)	Men (N = 1,274,287)	Women (N = 1,862,634)
	Model 1	Model 2	Model 3	Model 4	Model 5	Model 6
	Odds ratio (95% CI)	Odds ratio (95% CI)	Odds ratio (95% CI)	Odds ratio (95% CI)	Odds ratio (95% CI)	Odds ratio (95% CI)
Types of disability (ref. = No disability)						
Physical disability, moderate	0.854 (0.848, 0.859)	0.860 (0.854, 0.866)	0.799 (0.790, 0.808)	0.828 (0.808, 0.847)	0.895 (0.888, 0.901)	0.871 (0.865, 0.878)
Physical disability, severe	1.433 (1.419, 1.447)	1.934 (1.909, 1.959)	1.067 (1.043, 1.091)	1.049 (0.997, 1.103)	1.575 (1.558, 1.593)	2.061 (2.033, 2.089)
Visual disability, moderate	0.904 (0.892, 0.917)	0.997 (0.980, 1.015)	0.874 (0.851, 0.897)	0.917 (0.872, 0.963)	0.936 (0.921, 0.951)	1.017 (0.998, 1.036)
Visual disability, severe	1.127 (1.092, 1.163)	1.305 (1.264, 1.349)	0.751 (0.692, 0.815)	0.831 (0.733, 0.942)	1.230 (1.188, 1.273)	1.362 (1.316, 1.409)
Communication, moderate	0.883 (0.873, 0.894)	1.003 (0.990, 1.016)	0.742 (0.719, 0.765)	0.808 (0.764, 0.855)	0.908 (0.896, 0.920)	1.009 (0.995, 1.023)
Communication, severe	1.144 (1.120, 1.167)	1.287 (1.258, 1.318)	0.772 (0.733, 0.813)	0.899 (0.834, 0.968)	1.255 (1.227, 1.284)	1.353 (1.320, 1.387)
Mental disability	0.885 (0.867, 0.903)	1.435 (1.402, 1.469)	0.665 (0.626, 0.707)	0.827 (0.736, 0.930)	0.852 (0.833, 0.871)	1.469 (1.434, 1.505)
Developmental/Intellectual	1.150 (1.126, 1.174)	1.640 (1.606, 1.675)	0.615 (0.555, 0.682)	0.608 (0.521, 0.709)	1.161 (1.136, 1.187)	1.705 (1.669, 1.742)
Facial disability, moderate	0.495 (0.392, 0.625)	0.895 (0.721, 1.111)	0.412 (0.281, 0.603)	0.379 (0.199, 0.721)	0.552 (0.406, 0.751)	1.065 (0.838, 1.354)
Facial disability, severe	1.029 (0.867, 1.221)	1.004 (0.824, 1.224)	0.895 (0.672, 1.193)	1.070 (0.724, 1.581)	1.094 (0.880, 1.361)	0.994 (0.791, 1.251)
Epilepsy disability, moderate	0.940 (0.862, 1.025)	0.926 (0.845, 1.015)	0.499 (0.363, 0.685)	0.352 (0.206, 0.602)	0.983 (0.897, 1.078)	0.978 (0.890, 1.075)
Epilepsy disability, severe	1.000 (0.857, 1.167)	1.263 (1.072, 1.488)	0.370 (0.191, 0.717)		1.085 (0.920, 1.279)	1.377 (1.163, 1.632)
Internal disability, moderate	1.708 (1.664, 1.752)	1.941 (1.875, 2.008)	1.854 (1.779, 1.932)	1.962 (1.801, 2.137)	1.599 (1.548, 1.652)	1.960 (1.888, 2.034)
Internal disability, severe	2.372 (2.324, 2.421)	3.065 (2.985, 3.148)	2.141 (2.043, 2.244)	2.195 (1.994, 2.415)	2.514 (2.458, 2.573)	3.216 (3.128, 3.307)
Age	0.909 (0.908, 0.910)	0.783 (0.782, 0.783)	1.059 (1.057, 1.062)	0.891 (0.888, 0.894)	0.818 (0.817, 0.819)	0.759 (0.758, 0.759)
Age square	1.001 (1.001, 1.001)	1.002 (1.002, 1.002)	0.999 (0.999, 0.999)	1.001 (1.001, 1.001)	1.002 (1.001, 1.002)	1.002 (1.002, 1.002)
Healthcare coverage (ref. = NHI, employees)						
NHI, region	6.032 (5.978, 6.086)	5.572 (5.523, 5.621)	-	-	1.00	1.00
Medical aid	3.379 (3.370, 3.388)	2.995 (2.986, 3.004)	-	-	2.213 (2.193, 2.234)	1.957 (1.939, 1.975)
Income level (ref. = Highest income)						
Lowest income	1.952 (1.945, 1.959)	1.109 (1.105, 1.113)	2.868 (2.852, 2.884)	1.190 (1.181, 1.200)	1.304 (1.297, 1.311)	1.033 (1.029, 1.038)
2nd income	1.419 (1.413, 1.425)	0.836 (0.833, 0.840)	1.982 (1.970, 1.994)	0.837 (0.830, 0.844)	0.998 (0.992, 1.004)	0.852 (0.847, 0.856)
3rd income	1.206 (1.202, 1.211)	0.873 (0.870, 0.876)	1.478 (1.470, 1.486)	0.859 (0.852, 0.867)	0.981 (0.976, 0.986)	0.883 (0.880, 0.887)
4th income	1 .000(0.997, 1.004)	0.891 (0.888, 0.895)	1.087 (1.081, 1.092)	0.953 (0.944, 0.962)	0.947 (0.942, 0.951)	0.889 (0.885, 0.892)
Regions of residence (ref. = Metropolitan)						
Cities	0.737 (0.734, 0.740)	0.816 (0.813, 0.819)	0.616 (0.613, 0.619)	0.661 (0.656, 0.665)	0.871 (0.867, 0.876)	0.882 (0.878, 0.886)
Rural	0.801 (0.798, 0.803)	0.861 (0.858, 0.863)	0.709 (0.706, 0.712)	0.735 (0.731, 0.739)	0.906 (0.902, 0.910)	0.913 (0.910, 0.917)
Year (ref. = 2020)	0.678 (0.676, 0.680)	0.674 (0.673, 0.676)	0.607 (0.605, 0.609)	0.620 (0.617, 0.622)	0.734 (0.731, 0.736)	0.694 (0.692, 0.696)

The analytic models included year-fixed effect. NHI means National Health Insurance. ref means reference. All models are adjusted for age (as a continuous variable with its square term), sex, year (2019 vs. 2020), healthcare coverage type, income level (quintiles), and region of residence.

## Discussion

Despite the overall increase in health screening rates from 2012 to 2020, persons with disabilities consistently showed lower rates compared to those without disabilities. After adjusting for sex and age, the following subgroups of individuals with disabilities consistently showed lower health screening rates: severe physical, visual, severe communication, severe facial, epilepsy, mental, developmental, and internal disabilities. In contrast, those with moderate physical, communication, and facial disabilities exhibited rates comparable to those of individuals without disabilities. The disability-related disparity diminished with sex-age standardization and further adjustments for health coverage type, income quintile, and region of residence. In particular, moderate disparities across most disability types, except for internal disabilities, aligned with those of individuals without disabilities after adjusting for socioeconomic conditions. Furthermore, the disability-related disparity in health screening rates also differed by employment status.

Our findings are consistent with previous studies that identified lower participation in regular health check-ups [13, 14] and cancer screenings [7–12] among individuals with physical disabilities and cognitive impairments. Conversely, some studies did not find significant differences in breast and colorectal cancer screening rates among persons with physical disabilities [11, 22]. Our results, indicating significantly lower rates among those with severe physical disability but not moderate ones, suggest that the severity of disabilities may play a crucial role. Previous literatures has shown that severe disabilities elevate the risk of not receiving preventive health services [13, 19] due to barriers such as access difficulties, providers’ unwillingness and unpreparedness, and lack of support systems [16, 18, 28]. Additionally, compromised vital functions and limited functional reserve in patients with major organ disabilities contribute to a lower rate of health screening [29].

Individuals with intellectual and developmental disabilities also face significant health disparities due to limited access to the healthcare system [30] as well as avoidable premature mortality [31]. This population requires the support of health professionals who are knowledgeable and skilled to address their particular challenges and vulnerabilities in maintaining health [17]. Recent reviews have indicated that mental disorders are associated with reduced likelihood of receiving cancer and cardiovascular disease screenings, primarily due to the numerous barriers faced when accessing preventive care [32, 33]. Both provider and patient factors contribute to these barriers. Clinician-level factors include poor attention from healthcare providers, insufficient training, low mental health literacy, and negative attitudes. Patient-level factors encompass cognitive and social impairments, poor socio-economic conditions, and limited health literacy [32, 33].

Our findings demonstrated that adjusting for socio-economic conditions, such as the health coverage type and income level, reduced the disability-related disparity for the following subgroups: moderate visual, mental, intellectual, developmental, and severe epilepsy disabilities. This suggests that disability-related disparities are dependent on socio-economic conditions even with nationwide health insurance coverage. For example, although the epilepsy subgroup initially showed higher odds of not receiving health screenings, these odds became comparable to the individuals without disabilities after adjusting for socio-economic conditions. Epilepsy is associated with significant physical, mental, or social function limitations [34], acting as barriers to receiving health screening. Improving their socio-economic conditions may be essential in overcoming these barriers.

We also investigated health screening rates across disability subgroups by stratifying employment status. Notably, employed participants experienced smaller disability-related disparities than non-employed participants, although those with severe physical and internal disabilities still had lower screening rates compared to their non-disabled counterparts. This may be attributed to the GHSP mandate, which requires employers to provide regular health screenings for their employees [20]. Consequently, employers often contract with examiners and notify their employees to undergo the examination. Moreover, some employers use examination agencies that dispatch examiners to workplaces, enhancing accessibility for employees. Such active interventions may mitigate many of the disparities associated with disability.

This study has several limitations. First, while we evaluated health screening participation, we did not include detailed information on the specific types of health screenings conducted or their outcomes. For instance, the study does not distinguish whether blood pressure measurements were based on clinical visits or specific diagnostic tests, nor does it account for variations in how diabetes (e.g., HbA1c, point-of-care glucose, or fasting glucose) and dyslipidemia were assessed. This lack of detail restricts our ability to comprehensively evaluate the quality and completeness of health screenings. Second, given the large sample size, the study has increased statistical power, which can detect small differences that may lack clinical significance; therefore, the p-values should be interpreted with caution. Third, the study period overlaps with the COVID-19 pandemic, which may have affected health screening behaviors. During this time, health services often prioritized acute care while minimizing in-person visits. This situation may have led to lower screening rates due to postponed or canceled appointments. Crucially, these pandemic-related disruptions likely exacerbated pre-existing health disparities. Individuals with disabilities may have faced compounded barriers, including a greater perceived risk of infection in healthcare settings, potentially widening the screening gap observed in 2020. Nevertheless, the pandemic-related decline in screening participation in South Korea was found to be relatively moderate compared to the sharper decreases reported in other high-income countries, such as the United States, the United Kingdom, and Japan [35]. Fourth, the administrative data has inherent limitations regarding disability classification. The registration system adjudicates individuals with multiple conditions into a single final classification, precluding analysis of the compounded effects or interactions of co-occurring disabilities. Additionally, the binary severe/non-severe classification for mental and intellectual/developmental disabilities oversimplifies a wide spectrum of functional abilities, potentially masking important variations in healthcare access within these heterogeneous groups. Therefore, our findings for these specific disabilities need be interpreted with caution. Fifth, this study utilized a pooled cross-sectional dataset for the 2019–2020 regression analyses, treating each person-year as an independent observation. While this approach was necessary to accurately represent the biennial screening cycle, it does not account for the potential correlation of outcomes within the same individuals who were eligible in both years. Despite this, all analytical models were adjusted for the year to account for period-specific effects.

Nevertheless, the health screening rate remains low for those with severe physical and internal disabilities compared to the individuals without disabilities. For persons with internal disabilities, processes such as fasting, endoscopy, and drug administration may pose health risks that outweigh the benefits of the screenings with their complex health problem [36]. In addition, persons with severe physical disabilities may struggle to find examination facilities equipped to meet their specific needs, such as wheelchair-accessible scales, disability-specific height meters, portable electric lifts, and adjustable beds, compared to those with other types of disabilities, leading to indefinite delays in screenings [37]. Although the South Korean government began designating and supporting health screening centers for people with disabilities in 2018, our trend analysis did not show a clear, immediate acceleration in screening participation rates for this population post-implementation. A likely explanation for the lack of a strong national-level effect is the limited scale of the initiative. As previously noted, accessibility to these dedicated institutions remains a significant challenge, with only about 15 centers available nationwide [38]. Therefore, while these centers represent a crucial policy step, their current number appears insufficient to create a substantial impact on the national screening disparities observed in our study. This highlights the need for a significant expansion of such accessible infrastructure to translate policy intentions into measurable nationwide improvements.

The sex stratification analysis revealed more pronounced disability-related disparities for women with higher ORs compared to men. While previous studies have reported lower utilization rates of cancer and other screening services, as well as a reduced likelihood of engaging in health-promoting behaviors, for disabled women compared to their non-disabled counterparts, no studies have compared these disparities between men and women. Literature on disparities in the use of preventive services among women suggest that minority women have less education and information for self-care [39]. This implies that the generally lower socio-economic resources among women with disabilities, compared to their men counterparts, may exacerbate disability-related disparities. In particular, women with severe epilepsy and developmental disorders showed significantly lower health screening rates within the entire women population, whereas such differences were not observed within the men population. Difficulties in body control and reluctance to receive health screenings may contribute to these lower rates among women with developmental [40].

In conclusion, this study examined health screening rates among over three million South Koreans from 2012 to 2020. While the overall screening rate increased over time, individuals with disabilities—specifically those with severe physical, communication, severe facial, visual, internal, mental, developmental, and epilepsy impairments—consistently had lower screening rates than those without disabilities. Even after adjusting for various factors, individuals with internal and severe physical disabilities still showed a lower tendency to receive health screenings. Notably, this study also highlighted higher health screening rates among employed individuals, underscoring the importance of improving access to health screening institutions for unemployed or non-working persons with disabilities. Our findings emphasize the urgent need for inclusive healthcare practices and offer insights for policymakers and healthcare organizations aiming to address the unique challenges faced by this population.
