# Supplementary material for: Disability-Related Disparities in Preventive Healthcare Access in South Korea: Insights From National Health Insurance Data
Source: Int J Public Health. 2025 Nov 14;70:1608644. doi: 10.3389/ijph.2025.1608644 (PMC12660150; doi:10.3389/ijph.2025.1608644)
Supplement: Supplementary file 1 [file DataSheet1.pdf]

Supplementary materials

Table S1. Disability type and severity and categorization for this study (South Korea, 2012–2020)

Type	Classification	Severity	Categorization
Orthopedic impairment	Amputation, joint disorders, dysfunction of extremities, spine disorders, and other disorders such as deformities	M, S	Physical disability – M, S
Disability due to brain injury	Multiple disabilities due to brain injury	M, S	
Visual disability	Vision impairment and visual field defect	M, S	Visual disability – M, S
Hearing disability	Hearing impairment and balance disorder	M, S	Hearing/speech disability – M, S
Speech and language disability	Language and voice disorder	M, S	
Intellectual disability	IQ below 70	S	Intellectual/developmental disability –S
Autism spectrum disorder	Autism spectrum disorder such as childhood disintegrative disorders	S	
Mental disorder	Schizophrenia, schizoaffective disorder, bipolar affective disorder, recurrent depressive disorder, etc.	S	Mental disability – S
Disability due to renal failure	On dialysis or after kidney transplantation	M, S	Internal disability – M, S
Disability due to heart problems	Heart dysfunction that substantially limits major life activities	M, S	
Disability due to respiratory problems	Chronic or severe pulmonary dysfunction that substantially limits major life activities	M, S	
Disability due to liver disease	Chronic or severe liver dysfunction that substantially limits major life activities	M, S	
Disability due to ostomies	Stoma that substantially limits major life activities	M, S	
Disability due to facial disfigurement	Facial deformities such as scarring, depression, and thickening	M, S	Disability due to facial disfigurement – M, S
Disability due to epilepsy	Chronic or severe epilepsy that substantially limits major life activities	M, S	Epilepsy disability – M, S

M: moderate, S: severe. Disability type, classification, and severity are defined according to the disability criteria of Korean Ministry of Health and Welfare

Table S2. Age- and sex-adjusted health screening rate across different disability groups (South Korea, 2012–2020)

Disability status	2012	2013	2014	2015	2016	2017	2018	2019	2020
No disability	70.05	69.23	71.86	70.14	72.07	72.88	74.89	76.17	69.52
Any disability	63.25	63.12	65.39	63.15	64.61	64.54	65.05	66.14	59.43
Physical disability, moderate	71.74	70.88	72.95	70.74	72.74	72.52	73.99	75.07	68.60
Physical disability, severe	53.28	53.82	56.15	53.84	55.66	54.90	55.56	55.73	49.01
Visual disability, moderate	68.66	68.45	70.50	68.89	70.18	70.24	71.57	73.20	66.81
Visual disability, severe	55.61	55.33	58.39	58.02	58.76	58.33	59.11	61.66	53.86
Communication disability, moderate	69.30	68.52	70.01	68.55	70.44	71.98	71.84	73.95	67.58
Communication disability, severe	61.01	61.28	63.86	61.31	63.48	63.78	64.19	67.19	60.09
Mental disability	44.02	50.13	53.61	49.69	49.85	50.51	48.51	50.88	43.39
Developmental/Intellectual disability	43.41	45.57	48.75	45.93	46.74	46.43	45.48	46.25	39.97
Facial disability, moderate	74.67	78.96	71.21	77.03	70.52	74.24	73.04	81.24	76.09
Facial disability, severe	61.21	70.54	65.08	64.08	63.11	71.78	70.83	78.63	59.72
Epilepsy disability, moderate	58.01	62.56	68.50	61.88	60.48	60.50	57.53	60.55	53.95
Epilepsy disability, severe	60.42	56.13	64.25	60.88	52.19	57.97	44.10	57.00	44.66
Internal disability, moderate	55.90	54.33	57.60	55.12	55.61	56.47	56.51	57.97	53.73
Internal disability, severe	40.00	40.84	43.74	42.69	43.20	43.56	42.61	43.20	38.24

Figure S1. Age- and sex-adjusted health screening rate across different disability groups (South Korea, 2012–2020)

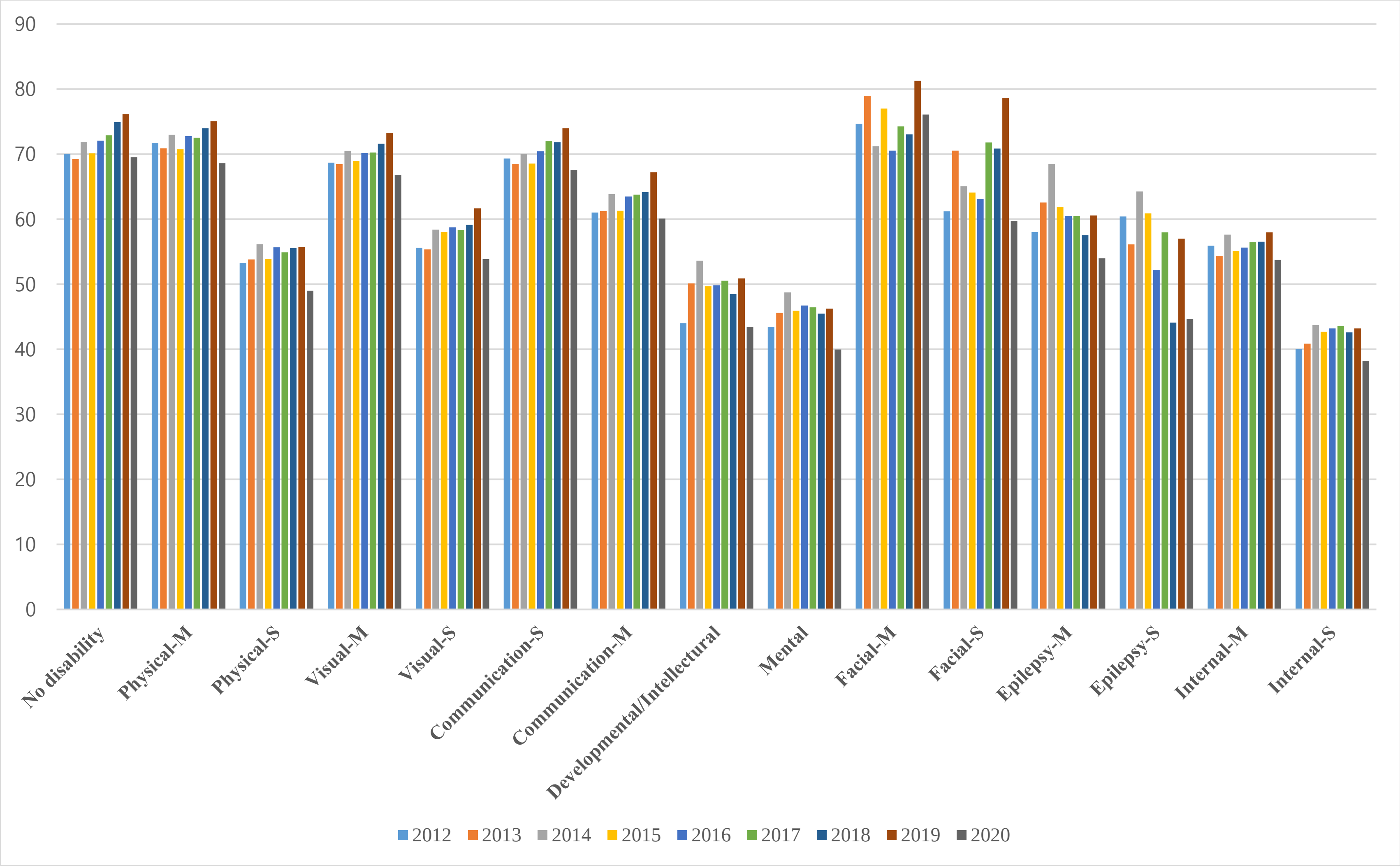

S: Severe disability, M: Moderate disability
